# Neurogenic Dysphagia: Peripheral and Central Neuromodulation

**DOI:** 10.3390/clinpract15090163

**Published:** 2025-09-04

**Authors:** Mario Stampanoni Bassi, Diego Centonze, Bledar Gjikolaj, Angelo Alito, Adriana Tisano, Rosario Marchese-Ragona, Domenico Antonio Restivo

**Affiliations:** 1 Unit of Neurology, IRCCS Neuromed, 86077 Pozzilli, Italy; m.stampanonibassi@gmail.com (M.S.B.); diego.centonze@neuromed.it (D.C.); bledargjikolaj@gmail.com (B.G.); 2 Department of System Medicine, Tor Vergata University, 00133 Rome, Italy; 3 Department of Biomedical, Dental Sciences and Morphological and Functional Images, University of Messina, 98125 Messina, Italy; alitoa@unime.it; 4 Department of Clinical and Experimental Medicine, University of Messina, 98125 Messina, Italy; atisano@unime.it; 5 Department of Neurosciences, University of Padova, 35121 Padova, Italy; rosario.marcheseragona@unipd.it

**Keywords:** dysphagia, neuromodulation, rehabilitation, swallowing

## Abstract

Dysphagia is a frequent and potentially life-threatening complication in patients with neurological disorders. Swallowing is a complex neurophysiological mechanism regulated by a widespread network of central nervous system regions. The control of swallowing functions requires the integrity of the central pattern generator located in the brainstem, the sensorimotor cortex, the basal ganglia, and the cerebellum, but also peripheral nerves and swallowing muscles. Neurological diseases affecting either central or peripheral components of this system commonly result in dysphagia. Despite its clinical relevance, the management of neurogenic dysphagia remains challenging. While rehabilitative strategies such as swallowing therapy currently represent the main treatment option, emerging evidence suggests that non-invasive central and peripheral neuromodulation techniques may provide adjunctive beneficial effects. Further research is warranted to better define their efficacy, optimal protocols, and long-term outcomes.

## 1. Introduction

Swallowing is an innate physiological mechanism that enables the ingestion of liquids and food without aspiration. It is characterized by a semi-automatic sensorimotor process that involves the muscles of the mouth, larynx, pharynx, and gastrointestinal tract [1]. Dysphagia, the disruption of the normal swallowing process, is a frequent and severe complication of many neurological disorders. It is associated with an increased risk of severe complications such as aspiration pneumonia, malnutrition, and prolonged hospitalization requiring invasive treatments. Dysphagia is also a leading cause of mortality in patients with neurological disorders [2,3].

An accurate diagnosis and early management of dysphagia can significantly improve quality of life, while also helping to delay life-threatening complications and the need for invasive therapeutic interventions [4].

However, the management of dysphagia remains challenging, with rehabilitation—specifically swallowing therapy—being the main therapeutic approach [5]. Conversely, invasive interventions, such as upper esophageal sphincter myotomy or percutaneous endoscopic gastrostomy (PEG), are reserved for selected cases [6]. Recently, non-invasive neuromodulation techniques, including peripheral electrical stimulation (PES), transcranial magnetic stimulation (TMS), and transcranial direct current stimulation (tDCS), have emerged as promising complementary approaches to rehabilitation [7,8,9]. Numerous studies suggest that these tools could be beneficial in treating dysphagia of different etiologies, including stroke, traumatic brain injuries, inflammatory diseases, and neuromuscular and neurodegenerative diseases.

## 2. Anatomy and Physiology of Swallowing

Swallowing is a complex physiological process that requires the coordinated activity of over fifty pairs of muscles in the lower face, larynx, pharynx, and esophagus [10]. This highly coordinated muscular activity ensures the safe passage of food and liquids from the oral cavity through the pharynx and esophagus into the stomach, preventing aspiration into the airway.

Between the end of the pharyngeal phase and the beginning of the esophageal phase, the upper esophageal sphincter (UES) relaxes and opens, allowing the bolus to pass into the esophagus. The UES is a high-pressure zone located between the pharynx and the cervical esophagus [11,12].

The physiological high-pressure zone of the UES corresponds in size and location to the tonically contracting striated cricopharyngeal (CP) muscle. The role of the UES is to protect against the reflux of food into the respiratory tract and prevent air from entering the digestive tract. However, from a clinical point of view, the oral and pharyngeal phases are generally considered a single phase. The oropharyngeal phase is more frequently impaired in neurological diseases [13]. This phase is characterized by complex changes in the morphology of the larynx and pharynx, ensuring protection of the airways and allowing the passage of the bolus through the upper esophageal sphincter (UES) [13].

Swallowing is regulated by a central pattern generator (CPG) located in the medulla oblongata. This center is bilaterally represented, and it very likely corresponds to the nucleus tractus solitarius (NTS) and the nucleus ambiguous (NA). CPG is composed of two groups of interneurons: the ventral and dorsal swallowing groups [10]. The two hemi-CPGs integrate peripheral afferent inputs, including sensory and gustatory fibers originating from the oropharyngeal mucosa, as well as proprioceptive feedback from muscles involved in swallowing. In addition, CPGs receive central inputs (i.e., ipsilateral and contralateral corticobulbar projections). Notably, studies using brain fMRI and PET have shown that voluntary swallowing activates a large bilateral network involving various cortical regions, the cerebellum, and subcortical nuclei [14]. The efferent pathways of the swallowing circuit comprise various cranial nerves, including the trigeminal (V), facial (VII), glossopharyngeal (IX), vagus (X), and hypoglossal (XII) nerves, each controlling specific muscle groups.

The opening of the UES marks the end of the oropharyngeal phase and is determined by relaxation of the cricopharyngeal (CP) muscle, elevation of the larynx, and the propulsive action of hypopharyngeal muscles, especially the inferior pharyngeal constrictor muscles [15,16,17]. Inhibition of the tonically activated CP during the hypopharyngeal phase is controlled by the CPGs and requires integration of both central and peripheral afferences [18,19].

The tonic inhibition of UES during the hypopharyngeal phase is controlled by afferent signals from the Xth cranial nerve and requires the integration of central and peripheral signals [18,19]. The CPG leads to pressure relaxation in the UES region, which effectively reduces the restrictive forces that impede bolus flow. Intraluminal bolus forces and extrinsic expansive traction forces exerted by the hyolaryngeal muscles (mylohyoid, anterior digastric, and posterior digastric), as well as changes in intrinsic tissue, induce UES opening following the pressure increase produced by the palate and larynx. If an impairment affects the competence of the palate and larynx, the ‘pressure chamber’ will not close [12].

Proper functioning of this complex neuroanatomical network is essential for functional swallowing and to prevent potentially life-threatening complications.

## 3. Pathophysiology of Neurogenic Dysphagia

Neurogenic dysphagia occurs when one or more components of the swallowing network (i.e., the CPG or its associated afferent and efferent pathways) are affected.

Therefore, neurogenic dysphagia represents a complex syndrome with multiple pathogenetic mechanisms and different clinical forms [20]. The type and severity of swallowing deficits vary according to the site of damage and extent of the pathological alterations [21]. In fact, studies using fiberoptic evaluation of swallowing (FEES) have identified different clinical phenotypes of dysphagia, which associate differently with underlying etiologies.

In stroke patients, the most frequent clinical abnormality is premature bolus spillage, which results from an oral phase deficit and may lead to preglutitive aspiration [22]. However, this sign is a nonspecific sign observed in neurogenic dysphagia from different etiologies. In patients with infratentorial stroke, a delayed swallowing reflex is typically observed and has been specifically associated with sensory deficits (i.e., pharyngeal hypoesthesia) that impair swallowing initiation [23]. Pharyngeal motility disorders (e.g., with bradykinesia and tremor) are characteristics of Parkinsonian syndromes, particularly PSP. In the early stages of PD, dysphagia is typically characterized by impairment of the oral phase. Conversely, in the late stages, pharyngeal phase impairment with impaired UES relaxation can also occur. Similarly, dysphagia associated with pharyngeal phase impairment can be observed in the early stages of Parkinsonian syndromes [18]. The presence of residuals in the valleculae and pyriform sinuses has been mainly associated with motor deficits in Parkinsonian syndromes and myositis [20]. However, in patients with PD, dysphagia is often exacerbated by drooling due to reduced saliva clearance resulting from diminished oro-facial movement [24].

Stroke represents the main cause of neurogenic dysphagia. Dysphagia represents a frequent complication of cerebrovascular disorders, being associated with prolonged hospitalization, increased mortality, and higher burden on healthcare systems [25,26,27]. Lesions directly involving the CPG, as in patients with lateral medullary infarction (i.e., Wallenberg syndrome), are associated with severe alteration of the oro-pharyngeal phase of swallowing and a higher risk of penetration and aspiration [28]. Dysphagia may also result from ischemic or hemorrhagic lesions of other brain areas, including frontal and insular cortex, basal ganglia, and cerebellum [29,30]. These regions are part of the swallowing network, processing sensorimotor information and providing afferent inputs to the CPGs.

Also, dysphagia is frequently observed in patients with multiple sclerosis (MS) and results from the presence of inflammatory demyelination and neurodegeneration affecting swallowing brain circuits [16]. Demyelinating lesions located in the brainstem and extensive involvement of the corticospinal tracts have been associated with severe MS dysphagia [6,16,31,32,33]. The prevalence of dysphagia is increased in MS patients with longer disease duration and higher disability. However, swallowing disorders can also be observed in newly diagnosed patients [34,35,36].

Moreover, dysphagia represents a severe complication of motor neuron diseases (e.g., amyotrophic lateral sclerosis, ALS). In patients with ALS, swallowing deficits may vary, reflecting the degree of both upper and lower motor neuron dysfunction [15]. Predominant upper motor neuron involvement has been associated with deficits in triggering swallowing and abnormal prolongation of the oropharyngeal phase due to impaired relaxation of the CP muscle responsible for the opening of the UES [32,37,38,39]. Furthermore, an isolated impairment of the UES opening, accompanied by a lack of EMG features and clinical signs/symptoms of lower motor neuron involvement at the bulbar level, has been considered an indicator of upper motor neuron involvement in the cortico-pharyngeal tract [32]. Conversely, degeneration of lower motor neurons is associated with weakness and atrophy of swallowing muscles, leading to impaired contraction and reduced efficiency of the oropharyngeal phase [40,41].

In Parkinson’s disease (PD) and other extrapyramidal disorders, including multiple system atrophy (MSA) and progressive supranuclear palsy (PSP), dysphagia has a profound impact on disease course. Evidence suggests that both dopaminergic and non-dopaminergic mechanisms may contribute to swallowing difficulties in PD. Brain fMRI studies showed that the basal ganglia are activated during swallowing [30]. Moreover, both levodopa and deep brain stimulation (DBS) may modulate the activity of swallowing brain networks, improving dysphagia in PD [42,43,44]. Other mechanisms include the involvement of non-dopaminergic brain areas; for example, the CPG may be directly affected by alpha-synuclein deposition [45]. In addition, post-mortem studies have shown alpha-synuclein also deposits in pharyngeal nerves and muscles, also indicating a peripheral component in PD dysphagia [46,47]. In idiopathic PD, slow swallowing, with reduced voluntary involvement of oral and/or submental muscles, is the most common finding. Severe dysphagia, with dysfunction of the reflex cough and impaired relaxation of the cricopharyngeal muscle, is observed in PD patients with longer disease duration and higher disability. Conversely, in patients with MSA and PSP, dysphagia is frequently observed from the early phases and characterized by impaired relaxation of the cricopharyngeal muscle [18,48].

Traumatic brain injury (TBI) constitutes a significant cause of neurogenic dysphagia. Individuals affected by TBI may experience either transient or persistent dysphagia, with the severity depending on the extent and localization of the cerebral damage [49,50]. Dysphagia is particularly frequent and severe among patients admitted to intensive care units, especially those who have undergone invasive mechanical ventilation or who develop critical illness neuropathy [51]. Moreover, swallowing disorders may arise from peripheral pathologies involving the nerves, the neuromuscular junction, or the muscles, with consequent impairment of both afferent inputs to and efferent outputs from the CPG.

Finally, aging is associated with a progressive decline in swallowing efficiency, primarily due to a slowing of the oropharyngeal phase, which renders swallowing more susceptible to dysfunction or injury from other causes. Accordingly, presbyphagia should be considered an important risk factor for the development of neurogenic dysphagia [52].

## 4. Rehabilitation and Neuromodulation

### 4.1. Exercise and Sensory Stimulation

The treatment of neurogenic dysphagia relies on an integrated approach that varies according to the severity of symptoms and the underlying neurological condition. Therapeutic strategies targeting the primary disease, by mitigating neuronal damage, as in stroke and MS, or by alleviating symptoms, as with dopaminergic therapy in PD, play a primary role in the management of swallowing problems. However, to date, no pharmacological intervention has demonstrated consistent efficacy in directly improving neurogenic dysphagia. Consequently, rehabilitation, combined with compensatory strategies, remains the main treatment option [53,54].

Therapy for dysphagia typically employs two main approaches: (1) repetitive swallowing exercises and targeted muscle-strengthening exercises, and (2) sensory stimulation techniques aimed at enhancing sensory input. These two complementary approaches aim at promoting effective swallowing by modulating the activity of the swallowing brain network [53,55].

Studies using TMS have demonstrated that swallowing exercises increase corticobulbar excitability [56]. Similarly, it has been shown that different sensory stimulation techniques (e.g., with ice or carbonated water) can enhance corticobulbar excitability by modulating afferent sensory inputs, which are conducted through peripheral nerves to the CPGs and to the cerebral cortex [57]. These effects are thought to result from long-term potentiation (LTP)-like neuroplastic changes within cortical and subcortical swallowing circuits [58].

### 4.2. Pharyngeal Electrical Stimulation (PES)

Neuromodulation techniques can potentially replicate or enhance the plasticity-related effects of rehabilitation and are largely employed in the treatment of various neurological symptoms [59,60]. Previous studies in neurogenic dysphagia have shown that promising methods include PES and non-invasive brain stimulation (NIBS), including techniques such as tDCS and repetitive TMS (rTMS).

PES is applied using intraluminal electrodes introduced with a nasogastric catheter and activates sensory fibers in the pharyngeal wall. Sensory afferent inputs travel with the IX and X cranial nerves and are transmitted to the NTS and to other brain regions (including subcortical and cortical areas). High-frequency stimulation (5 Hz) at 75% of maximum tolerated intensity for 10–20 min promotes neuroplastic changes in swallowing brain networks and is associated with a significant increase in corticobulbar excitability in healthy subjects and in patients with neurogenic dysphagia [32,61].

Most PES studies have been conducted in patients with post-stroke dysphagia [61,62,63,64]. In patients who had suffered a stroke, daily application of PES for three consecutive days improved airway protection compared with the control group. It also reduced aspiration and improved feeding status, resulting in shorter hospital stays [62]. Another study evaluating the long-term effects of PES on post-stroke dysphagia demonstrated that swallowing was recovered more quickly in the first two weeks after stimulation [64]. A meta-analysis of PES treatment for post-stroke dysphagia found that PES is related to decreased radiological aspiration and enhanced dysphagia, potentially reducing the length of hospital stay [63]. Another RCT enrolled 162 patients with dysphagia associated with subacute ischemic or hemorrhagic stroke. PES or sham stimulation was provided over three consecutive days. The primary outcome was swallowing efficacy after two weeks. The secondary outcomes included dysphagia severity, functional quality of life, and the occurrence of serious adverse events at 6 and 12 weeks. The results showed that PES was safe, but there was no significant improvement in aspiration test scores compared to the placebo. However, these results may have been influenced by confounding factors, including patients with mild dysphagia, under-treatment with PES, and active stimulation in the control group [65]. In another pilot study involving 30 tracheotomized patients with dysphagia and more severe strokes, PES enhanced the remission of dysphagia in 75% of patients, consequently enabling decannulation [66].

PES has also been applied in dysphagic patients with MS [32]. It has been reported that pharyngeal stimulation for 5 consecutive days improved swallowing function and reduced the risk of aspiration [32]. Finally, evidence suggests that PES constitutes a promising intervention for neurogenic dysphagia resulting from different underlying pathologies, including TBI and critical illness polyneuropathy [65].

### 4.3. Non-Invasive Brain Stimulation (NIBS)

The rationale of the application of NIBS in rehabilitation settings has been aimed at inducing prolonged effects on the neural network. It has been demonstrated that NBI techniques are able to modulate synaptic connectivity by producing long-term changes in cortical excitability, like long-term potentiation and long-term depression, which are considered relevant mechanisms of plastic reorganization [59,67,68]. The amount and the duration of the induced neurophysiological changes depend on the stimulation intensity and duration [67]. NIBS techniques include transcranial direct current stimulation (tDCS), repetitive transcranial magnetic stimulation (rTMS), and theta burst stimulation (TBS), all of which can be used to induce long-lasting changes in cortical excitability [59,68,69].

While rTMS shows promise for improving post-stroke dysphagia, current evidence remains limited and inconclusive due to significant methodological flaws in existing systematic reviews and meta-analyses [70].

tDCS employs weak electrical currents to induce persistent excitability changes in the human brain cortex [67,69]. The resulting effects depend on the polarity of stimulation [69]. Anodal tDCS depolarizes cortical neurons and induces LTP-like effects. It has been demonstrated that anodal tDCS can increase the excitability of the pharyngeal motor cortex and enhance the sucking activity of a liquid bolus in healthy subjects [71,72].

Different TMS protocols can induce a persistent increase or decrease in cortical excitability. Low-frequency (1 Hz) rTMS of the pharyngeal motor cortex induced a long-lasting suppression of pharyngeal MEPs resembling long-term depression (LTD) plasticity phenomena and altered normal swallowing in healthy subjects [73]. Conversely, specific TMS protocols, including high-frequency (>5 Hz) rTMS and intermittent TBS (iTBS), can induce a long-lasting increase in corticobulbar excitability and have been associated with long-term potentiation (LTP)-like effects [74]. iTBS and high-frequency rTMS of the pharyngeal motor cortex can restore cortico-pharyngeal excitability and swallowing impairment induced by 1 Hz rTMS [72]. Notably, similar findings have been reported with cerebellar rTMS [75,76,77].

Several studies have examined the role of NIBS in the treatment of neurogenic dysphagia. Most studies focused on post-stroke dysphagia. RCTs and meta-analyses of RCTs have shown that rTMS/TBS and anodal tDCS promoted recovery of swallowing function in patients with post-stroke dysphagia [78,79,80,81,82].

Studies have demonstrated that NIBS of the pharyngeal motor cortex exerts beneficial effects both in patients with cortical lesions and in those with brainstem and cerebellar involvement [83,84].

Notably, the effects are mediated by the potentiation of corticobulbar excitability, promoting reorganization of swallowing networks. Regarding the site of stimulation, it has been shown that both unilateral tDCS (applied either to the affected or the unaffected hemisphere) or bilateral tDCS induce a significant improvement in post-stroke dysphagia [78,79]. Also, rTMS has been applied using different protocols in post-stroke dysphagia [84]. Both unilateral, bilateral, and cerebellar stimulation have been associated with improvement in swallowing function and reduced complications [85,86]. However, the best stimulation protocol and site of stimulation according to the type of dysphagia have not been defined.

NIBS has also been explored in other types of neurogenic dysphagia, particularly in MS, Parkinsonian syndromes, and TBI.

Two previous studies have reported beneficial effects of anodal TDCS of the pharyngeal motor cortex in patients with MS [32,87]. Particularly, it has been shown that in severely dysphagic MS patients with bulbar lesions, anodal tDCS of the pharyngeal motor cortex significantly increased cricopharyngeal excitability and reduced the risk of aspiration [33].

Finally, it has been reported that rTMS and anodal tDCS may significantly improve swallowing deficits in patients with neurodegenerative conditions, including PD and PSP [88], and also in elderly patients with primary and secondary dysphagia [89].

## 5. Conclusions

Neurogenic dysphagia remains a major clinical concern due to its impact on morbidity and mortality in patients with neurological disorders. Recent advances in non-invasive neuromodulation techniques offer encouraging prospects for improving treatment outcomes. Current evidence, primarily derived from studies in post-stroke patients, supports the use of PES, rTMS, and tDCS for the management of post-stroke dysphagia.

However, the European Stroke Organization and the European Society for Swallowing Disorders (ESO-ESSD) guideline for the diagnosis and treatment of post-stroke dysphagia [90] recommends that neurostimulation treatment should preferably be conducted within a clinical trial setting. Quality of evidence: low. Strength of recommendation: Strong for intervention (Recommendation 19). Moreover, for patients with post-stroke dysphagia, they suggest the use of rTMS, TES, tDCS, and PES as an adjunct to conventional dysphagia treatments to improve swallowing function. Quality of evidence: Moderate. Strength of recommendation: Weak for intervention (Recommendation 20). Nevertheless, further clinical trials are necessary to establish standardized protocols and assess the long-term efficacy of these interventions.

## Data Availability

The data presented in this study are available within this article.

## Abbreviations

The following abbreviations are used in this manuscript:

ALS	Amyotrophic lateral sclerosis
CP	Cricopharyngeal
CPG	Central pattern generator
DBS	Deep brain stimulation
FEES	Fiberoptic evaluation of swallowing
iTMS	Intermittent TBS
LTD	Long-term depression
LTP	Long-term potentiation
MS	Multiple sclerosis
MSA	Multiple system atrophy
NA	Nucleus ambiguous
NIBS	Non-invasive brain stimulation
NTS	Nucleus tractus solitarius
PD	Parkinson’s disease
PEG	Percutaneous endoscopic gastrostomy
PES	Peripheral electrical stimulation
PSP	Progressive supranuclear palsy
rTMS	Repetitive TMS
TBI	Traumatic brain injury
TBS	Theta burst stimulation
tDCS	Transcranial direct current stimulation
TMS	Transcranial magnetic stimulation
UES	Upper esophageal sphincter

## References

[1] Matsuo K., Palmer J.B. (2008). Anatomy and physiology of feeding and swallowing: Normal and abnormal. Phys. Med. Rehabil. Clin. N. Am..

[2] Dziewas R., Allescher H.-D., Aroyo I., Bartolome G., Beilenhoff U., Bohlender J., Breitbach-Snowdon H., Fheodoroff K., Glahn J., Heppner H.-J. (2021). Diagnosis and treatment of neurogenic dysphagia—S1 guideline of the German Society of Neurology. Neurol. Res. Pract..

[3] Coelho P.M., Almeida P.L., Carmezim I., Silva A., Evangelista R., Dinis C., Martins T., Torres A., Gomes A., Caldas J. (2024). Mortality and Pulmonary Complications of Post-stroke Dysphagia: A Casuistic Review of an Acute Stroke Unit. Cureus.

[4] Aydogdu I., Kiylioglu N., Tarlaci S., Tanriverdi Z., Alpaydin S., Acarer A., Baysal L., Arpaci E., Yuceyar N., Secil Y. (2015). Diagnostic value of “dysphagia limit” for neurogenic dysphagia: 17years of experience in 1278 adults. Clin. Neurophysiol..

[5] Nakao-Kato M., Rathore F. (2023). An Overview Of The Management And Rehabilitation of Dysphagia. J. Pak. Med. Assoc..

[6] Restivo D.A., Marchese-Ragona R., Patti F., Solaro C., Maimone D., Zappalá G., Pavone A. (2011). Botulinum toxin improves dysphagia associated with multiple sclerosis. Eur. J. Neurol..

[7] Wang L., Shi A., Xue H., Li Q., Wang J., Yang H., Hong H., Lu Q., Cheng J. (2023). Efficacy of Transcranial Direct Current Stimulation Combined with Conventional Swallowing Rehabilitation Training on Post-stroke Dysphagia. Dysphagia.

[8] Leonardi G., Ciurleo R., Cucinotta F., Fonti B., Borzelli D., Costa L., Tisano A., Portaro S., Alito A. (2022). The role of brain oscillations in post-stroke motor recovery: An overview. Front. Syst. Neurosci..

[9] Restivo D.A., Hamdy S. (2018). Pharyngeal electrical stimulation device for the treatment of neurogenic dysphagia: Technology update. Med. Devices Evid. Res..

[10] Jean A. (2001). Brain stem control of swallowing: Neuronal network and cellular mechanisms. Physiol. Rev..

[11] Omari T.I., Wiklendt L., Dinning P., Costa M., Rommel N., Cock C. (2014). Upper esophageal sphincter mechanical states analysis: A novel methodology to describe UES relaxation and opening. Front. Syst. Neurosci..

[12] Omari T.I., Maclean J.C.F., Cock C., McCulloch T.M., Nativ-Zeltzer N., O’Rourke A.K., Szczesniak M.M., Wu P.I., Allen J., Aoyagi Y. (2025). Defining Pharyngeal and Upper Esophageal Sphincter Disorders on High-Resolution Manometry-Impedance: The Leuven Consensus. Neurogastroenterol. Motil..

[13] Logemann J.A. (1999). Do we know what is normal and abnormal airway protection?. Dysphagia.

[14] Hamdy S., Rothwell J.C., Brooks D.J., Bailey D., Aziz Q., Thompson D.G. (1999). Identification of the cerebral loci processing human swallowing with H2(15)O PET activation. J. Neurophysiol..

[15] Restivo D.A., Casabona A., Nicotra A., Zappia M., Elia M., Romano M.C., Alfonsi E., Marchese-Ragona R. (2013). ALS dysphagia pathophysiology: Differential botulinum toxin response. Neurology.

[16] Restivo D.A., Quartarone A., Bruschetta A., Alito A., Milardi D., Marchese-Ragona R., Iezzi E., Peter S., Centonze D., Stampanoni Bassi M. (2024). Dysphagia in multiple sclerosis: Pathophysiology, assessment, and management-an overview. Front. Neurol..

[17] Ramaswamy A.T., Martell P., Azevedo R., Belafsky P. (2021). The upper esophageal sphincter: Anatomy and physiology. Ann. Esophagus.

[18] Alfonsi E., Versino M., Merlo I.M., Pacchetti C., Martignoni E., Bertino G., Moglia A., Tassorelli C., Nappi G. (2007). Electrophysiologic patterns of oral-pharyngeal swallowing in parkinsonian syndromes. Neurology.

[19] Miller A.J. (1986). Neurophysiological basis of swallowing. Dysphagia.

[20] Warnecke T., Labeit B., Schroeder J., Reckels A., Ahring S., Lapa S., Claus I., Muhle P., Suntrup-Krueger S., Dziewas R. (2021). Neurogenic Dysphagia: Systematic Review and Proposal of a Classification System. Neurology.

[21] Cheng I., Hamad A., Sasegbon A., Hamdy S. (2022). Advances in the Treatment of Dysphagia in Neurological Disorders: A Review of Current Evidence and Future Considerations. Neuropsychiatr. Dis. Treat..

[22] Shaker R., Geenen J.E. (2011). Management of Dysphagia in stroke patients. Gastroenterol. Hepatol..

[23] Labeit B., Jung A., Ahring S., Oelenberg S., Muhle P., Roderigo M., Wenninger F., von Itter J., Claus I., Warnecke T. (2023). Relationship between post-stroke dysphagia and pharyngeal sensory impairment. Neurol. Res. Pract..

[24] Isaacson J., Patel S., Torres-Yaghi Y., Pagán F. (2020). Sialorrhea in Parkinson’s Disease. Toxins.

[25] Banda K.J., Chu H., Kang X.L., Liu D., Pien L.-C., Jen H.-J., Hsiao S.-T.S., Chou K.-R. (2022). Prevalence of dysphagia and risk of pneumonia and mortality in acute stroke patients: A meta-analysis. BMC Geriatr..

[26] Huang Z.X., Gu H.Q., Yang X., Wang C.J., Wang Y.J., Li Z.X. (2021). Risk factors for in-hospital mortality among acute ischemic stroke patients in China: A nationwide prospective study. Neurol. Res..

[27] Labeit B., Kremer A., Muhle P., Claus I., Warnecke T., Dziewas R., Suntrup-Krueger S. (2023). Costs of post-stroke dysphagia during acute hospitalization from a health-insurance perspective. Eur. Stroke J..

[28] Nakao M., Oshima F., Maeno Y., Izumi S. (2019). Disruption of the Obligatory Swallowing Sequence in Patients with Wallenberg Syndrome. Dysphagia.

[29] Jones C.A., Colletti C.M., Ding M.-C. (2020). Post-stroke Dysphagia: Recent Insights and Unanswered Questions. Curr. Neurol. Neurosci. Rep..

[30] Suzuki M., Asada Y., Ito J., Hayashi K., Inoue H., Kitano H. (2003). Activation of cerebellum and basal ganglia on volitional swallowing detected by functional magnetic resonance imaging. Dysphagia.

[31] Alfonsi E., Bergamaschi R., Cosentino G., Ponzio M., Montomoli C., Restivo D.A., Brighina F., Ravaglia S., Prunetti P., Bertino G. (2013). Electrophysiological patterns of oropharyngeal swallowing in multiple sclerosis. Clin. Neurophysiol..

[32] Restivo D.A., Casabona A., Centonze D., Marchese-Ragona R., Maimone D., Pavone A. (2013). Pharyngeal electrical stimulation for dysphagia associated with multiple sclerosis: A pilot study. Brain Stimul..

[33] Restivo D.A., Alfonsi E., Casabona A., Hamdy S., Tassorelli C., Panebianco M., Marchese-Ragona R., Quartarone A., Centonze D., Pavone A. (2019). A pilot study on the efficacy of transcranial direct current stimulation applied to the pharyngeal motor cortex for dysphagia associated with brainstem involvement in multiple sclerosis. Clin. Neurophysiol..

[34] Calcagno P., Ruoppolo G., Grasso M.G., De Vincentiis M., Paolucci S. (2002). Dysphagia in multiple sclerosis—Prevalence and prognostic factors. Acta Neurol. Scand..

[35] Grasso M.G., Gamberini G., Patti F., D’Amico E., Bergamaschi R., Berra E., Giusti A., Cuccaro A., Messmer Uccelli M., Solaro C. (2021). The Dysphagia in Multiple Sclerosis Questionnaire Correlates with Fiber-Optic Endoscopic Examination for Detecting Swallowing Deficits in MS. Dysphagia.

[36] Solaro C., Cuccaro A., Gamberini G., Patti F., D’Amico E., Bergamaschi R., Berra E., Giusti A., Rezzani C., Messmer Uccelli M. (2020). Prevalence of dysphagia in a consecutive cohort of subjects with MS using fibre-optic endoscopy. Neurol. Sci..

[37] Ertekin C., Kiylioglu N., Tarlaci S., Turman A.B., Secil Y., Aydogdu I. (2001). Voluntary and reflex influences on the initiation of swallowing reflex in man. Dysphagia.

[38] Kern M.K., Jaradeh S., Arndorfer R.C., Shaker R. (2001). Cerebral cortical representation of reflexive and volitional swallowing in humans. Am. J. Physiol. Gastrointest. Liver Physiol..

[39] Martin R.E., Goodyear B.G., Gati J.S., Menon R.S. (2001). Cerebral Cortical Representation of Automatic and Volitional Swallowing in Humans. J. Neurophysiol..

[40] Cosentino G., Alfonsi E., Mainardi L., Alvisi E., Brighina F., Valentino F., Fierro B., Sandrini G., Bertino G., Berlangieri M. (2017). The importance of the reproducibility of oropharyngeal swallowing in amyotrophic lateral sclerosis. An electrophysiological study. Clin. Neurophysiol..

[41] Kawai S., Tsukuda M., Mochimatsu I., Enomoto H., Kagesato Y., Hirose H., Kuroiwa Y., Suzuki Y. (2003). A study of the early stage of Dysphagia in amyotrophic lateral sclerosis. Dysphagia.

[42] Hunter P.C., Crameri J., Austin S., Woodward M.C., Hughes A.J. (1997). Response of parkinsonian swallowing dysfunction to dopaminergic stimulation. J. Neurol. Neurosurg. Psychiatry.

[43] Troche M.S., Brandimore A.E., Foote K.D., Okun M.S. (2013). Swallowing and deep brain stimulation in Parkinson’s disease: A systematic review. Park. Relat. Disord..

[44] Warnecke T., Hamacher C., Oelenberg S., Dziewas R. (2014). Off and on state assessment of swallowing function in Parkinson’s disease. Park. Relat. Disord..

[45] Braak H., Del Tredici K., Bratzke H., Hamm-Clement J., Sandmann-Keil D., Rüb U. (2002). Staging of the intracerebral inclusion body pathology associated with idiopathic Parkinson’s disease (preclinical and clinical stages). J. Neurol..

[46] Mu L., Sobotka S., Chen J., Su H., Sanders I., Adler C.H., Shill H.A., Caviness J.N., Samanta J.E., Beach T.G. (2012). Altered pharyngeal muscles in Parkinson disease. J. Neuropathol. Exp. Neurol..

[47] Mu L., Sobotka S., Chen J., Su H., Sanders I., Nyirenda T., Adler C.H., Shill H.A., Caviness J.N., Samanta J.E. (2013). Parkinson disease affects peripheral sensory nerves in the pharynx. J. Neuropathol. Exp. Neurol..

[48] Restivo D.A., Palmeri A., Marchese-Ragona R. (2002). Botulinum toxin for cricopharyngeal dysfunction in Parkinson’s disease. N. Engl. J. Med..

[49] Lee W.K., Yeom J., Lee W.H., Seo H.G., Oh B.M., Han T.R. (2016). Characteristics of Dysphagia in Severe Traumatic Brain Injury Patients: A Comparison With Stroke Patients. Ann. Rehabil. Med..

[50] Won S.Y., Krieger S., Dubinski D., Gessler F., Behmanesh B., Freiman T.M., Konczalla J., Seifert V., Lapa S. (2021). Neurogenic Dysphagia in Subdural Hematoma. Front. Neurol..

[51] Ponfick M., Linden R., Nowak D.A. (2015). Dysphagia--a common, transient symptom in critical illness polyneuropathy: A fiberoptic endoscopic evaluation of swallowing study*. Crit. Care Med..

[52] Labeit B., Muhle P., von Itter J., Slavik J., Wollbrink A., Sporns P., Rusche T., Ruck T., Hüsing-Kabar A., Gellner R. (2022). Clinical determinants and neural correlates of presbyphagia in community-dwelling older adults. Front. Aging Neurosci..

[53] Logemann J.A. (1994). Rehabilitation of oropharyngeal swallowing disorders. Acta Otorhinolaryngol. Belg..

[54] Huckabee M.-L., Macrae P. (2014). Rethinking Rehab: Skill-Based Training for Swallowing Impairment. Perspect. Swallowing Swallowing Disord. (Dysphagia).

[55] Sasegbon A., Cheng I., Hamdy S. (2025). The neurorehabilitation of post-stroke dysphagia: Physiology and pathophysiology. J. Physiol..

[56] Gallas S., Marie J.P., Leroi A.M., Verin E. (2009). Impact of swallowing and ventilation on oropharyngeal cortical representation. Respir. Physiol. Neurobiol..

[57] Alvarez-Berdugo D., Rofes L., Casamitjana J.F., Padrón A., Quer M., Clavé P. (2016). Oropharyngeal and laryngeal sensory innervation in the pathophysiology of swallowing disorders and sensory stimulation treatments. Ann. N. Y. Acad. Sci..

[58] Michou E., Hamdy S., Harris M., Vania A., Dick J., Kellett M., Rothwell J. (2014). Characterization of corticobulbar pharyngeal neurophysiology in dysphagic patients with Parkinson’s disease. Clin. Gastroenterol. Hepatol..

[59] Ziemann U., Paulus W., Nitsche M.A., Pascual-Leone A., Byblow W.D., Berardelli A., Siebner H.R., Classen J., Cohen L.G., Rothwell J.C. (2008). Consensus: Motor cortex plasticity protocols. Brain Stimul..

[60] Sasegbon A., Cheng I., Zhang M., Hamdy S. (2020). Advances in the Use of Neuromodulation for Neurogenic Dysphagia: Mechanisms and Therapeutic Application of Pharyngeal Electrical Stimulation, Transcranial Magnetic Stimulation, and Transcranial Direct Current Stimulation. Am. J. Speech Lang. Pathol..

[61] Fraser C., Power M., Hamdy S., Rothwell J., Hobday D., Hollander I., Tyrell P., Hobson A., Williams S., Thompson D. (2002). Driving plasticity in human adult motor cortex is associated with improved motor function after brain injury. Neuron.

[62] Jayasekeran V., Singh S., Tyrrell P., Michou E., Jefferson S., Mistry S., Gamble E., Rothwell J., Thompson D., Hamdy S. (2010). Adjunctive functional pharyngeal electrical stimulation reverses swallowing disability after brain lesions. Gastroenterology.

[63] Scutt P., Lee H., Hamdy S., Bath P. (2015). Pharyngeal Electrical Stimulation for Treatment of Poststroke Dysphagia: Individual Patient Data Meta-Analysis of Randomised Controlled Trials. Stroke Res. Treat..

[64] Vasant D.H., Michou E., O’Leary N., Vail A., Mistry S., Hamdy S. (2016). Pharyngeal Electrical Stimulation in Dysphagia Poststroke: A Prospective, Randomized Single-Blinded Interventional Study. Neurorehabilit. Neural Repair.

[65] Bath P.M., Scutt P., Love J., Clavé P., Cohen D., Dziewas R., Iversen H.K., Ledl C., Ragab S., Soda H. (2016). Pharyngeal Electrical Stimulation for Treatment of Dysphagia in Subacute Stroke: A Randomized Controlled Trial. Stroke.

[66] Suntrup S., Marian T., Schröder J.B., Suttrup I., Muhle P., Oelenberg S., Hamacher C., Minnerup J., Warnecke T., Dziewas R. (2015). Electrical pharyngeal stimulation for dysphagia treatment in tracheotomized stroke patients: A randomized controlled trial. Intensive Care Med..

[67] Nitsche M.A., Paulus W. (2000). Excitability changes induced in the human motor cortex by weak transcranial direct current stimulation. J. Physiol..

[68] Siebner H.R., Rothwell J. (2003). Transcranial magnetic stimulation: New insights into representational cortical plasticity. Exp. Brain Res..

[69] Nitsche M.A., Fricke K., Henschke U., Schlitterlau A., Liebetanz D., Lang N., Henning S., Tergau F., Paulus W. (2003). Pharmacological modulation of cortical excitability shifts induced by transcranial direct current stimulation in humans. J. Physiol..

[70] Georgiou A.M., Phylactou P., Kambanaros M. (2024). The effectiveness of transcranial magnetic stimulation for dysphagia in stroke patients: An umbrella review of systematic reviews and meta-analyses. Front. Human. Neurosci..

[71] Cosentino G., Alfonsi E., Brighina F., Fresia M., Fierro B., Sandrini G., Schindler A., Valentino F., Fontana D., Priori A. (2014). Transcranial direct current stimulation enhances sucking of a liquid bolus in healthy humans. Brain Stimul..

[72] Jefferson S., Mistry S., Singh S., Rothwell J., Hamdy S. (2009). Characterizing the application of transcranial direct current stimulation in human pharyngeal motor cortex. Am. J. Physiol. Gastrointest. Liver Physiol..

[73] Mistry S., Verin E., Singh S., Jefferson S., Rothwell J.C., Thompson D.G., Hamdy S. (2007). Unilateral suppression of pharyngeal motor cortex to repetitive transcranial magnetic stimulation reveals functional asymmetry in the hemispheric projections to human swallowing. J. Physiol..

[74] Michou E., Raginis-Zborowska A., Watanabe M., Lodhi T., Hamdy S. (2016). Repetitive Transcranial Magnetic Stimulation: A Novel Approach for Treating Oropharyngeal Dysphagia. Curr. Gastroenterol. Rep..

[75] Jayasekeran V., Rothwell J., Hamdy S. (2011). Non-invasive magnetic stimulation of the human cerebellum facilitates cortico-bulbar projections in the swallowing motor system. Neurogastroenterol. Motil..

[76] Vasant D.H., Michou E., Mistry S., Rothwell J.C., Hamdy S. (2015). High-frequency focal repetitive cerebellar stimulation induces prolonged increases in human pharyngeal motor cortex excitability. J. Physiol..

[77] Sasegbon A., Watanabe M., Simons A., Michou E., Vasant D.H., Magara J., Bath P.M., Rothwell J., Inoue M., Hamdy S. (2019). Cerebellar repetitive transcranial magnetic stimulation restores pharyngeal brain activity and swallowing behaviour after disruption by a cortical virtual lesion. J. Physiol..

[78] Cheng I., Sasegbon A., Hamdy S. (2021). Effects of Neurostimulation on Poststroke Dysphagia: A Synthesis of Current Evidence From Randomized Controlled Trials. Neuromodulation.

[79] Marchina S., Pisegna J.M., Massaro J.M., Langmore S.E., McVey C., Wang J., Kumar S. (2021). Transcranial direct current stimulation for post-stroke dysphagia: A systematic review and meta-analysis of randomized controlled trials. J. Neurol..

[80] Pisegna J.M., Kaneoka A., Pearson W.G., Kumar S., Langmore S.E. (2016). Effects of non-invasive brain stimulation on post-stroke dysphagia: A systematic review and meta-analysis of randomized controlled trials. Clin. Neurophysiol..

[81] Wang T., Dong L., Cong X., Luo H., Li W., Meng P., Wang Q. (2021). Comparative efficacy of non-invasive neurostimulation therapies for poststroke dysphagia: A systematic review and meta-analysis. Neurophysiol. Clin..

[82] Zhao N., Sun W., Xiao Z., Fan C., Zeng B., Xu K., Liao M., Lu W. (2022). Effects of Transcranial Direct Current Stimulation on Poststroke Dysphagia: A Systematic Review and Meta-analysis of Randomized Controlled Trials. Arch. Phys. Med. Rehabil..

[83] Khedr E.M., Abo-Elfetoh N. (2010). Therapeutic role of rTMS on recovery of dysphagia in patients with lateral medullary syndrome and brainstem infarction. J. Neurol. Neurosurg. Psychiatry.

[84] Labeit B., Michou E., Trapl-Grundschober M., Suntrup-Krueger S., Muhle P., Bath P.M., Dziewas R. (2024). Dysphagia after stroke: Research advances in treatment interventions. Lancet Neurol..

[85] Rao J., Li F., Zhong L., Wang J., Peng Y., Liu H., Wang P., Xu J. (2022). Bilateral Cerebellar Intermittent Theta Burst Stimulation Combined With Swallowing Speech Therapy for Dysphagia After Stroke: A Randomized, Double-Blind, Sham-Controlled, Clinical Trial. Neurorehabilit. Neural Repair.

[86] Zhong L., Rao J., Wang J., Li F., Peng Y., Liu H., Zhang Y., Wang P. (2021). Repetitive Transcranial Magnetic Stimulation at Different Sites for Dysphagia After Stroke: A Randomized, Observer-Blind Clinical Trial. Front. Neurol..

[87] Cosentino G., Gargano R., Bonura G., Realmuto S., Tocco E., Ragonese P., Gangitano M., Alfonsi E., Fierro B., Brighina F. (2018). Anodal tDCS of the swallowing motor cortex for treatment of dysphagia in multiple sclerosis: A pilot open-label study. Neurol. Sci..

[88] Khedr E.M., Mohamed K.O., Soliman R.K., Hassan A.M.M., Rothwell J.C. (2019). The Effect of High-Frequency Repetitive Transcranial Magnetic Stimulation on Advancing Parkinson’s Disease with Dysphagia: Double Blind Randomized Clinical Trial. Neurorehabilit. Neural Repair.

[89] Cosentino G., Tassorelli C., Prunetti P., Bertino G., De Icco R., Todisco M., Di Marco S., Brighina F., Schindler A., Rondanelli M. (2020). Anodal transcranial direct current stimulation and intermittent theta-burst stimulation improve deglutition and swallowing reproducibility in elderly patients with dysphagia. Neurogastroenterol. Motil..

[90] Dziewas R., Michou E., Trapl-Grundschober M., Lal A., Arsava E.M., Bath P.M., Clavé P., Glahn J., Hamdy S., Pownall S. (2021). European Stroke Organisation and European Society for Swallowing Disorders guideline for the diagnosis and treatment of post-stroke dysphagia. Eur. Stroke J..

